# Effect of cooking and smoking methods on proximate composition, lipid oxidation and mineral contents of *Polypterus bichir bichir* fish from far-north region of Cameroon

**DOI:** 10.1016/j.heliyon.2022.e10921

**Published:** 2022-10-03

**Authors:** Noel Tenyang, Ludovine Ateufack Mawamba, Roger Ponka, Abazidi Mamat, Bernard Tiencheu, Hilaire Macaire Womeni

**Affiliations:** a University of Maroua, Faculty of Science, Department of Biological Science, P. O. Box 814, Maroua, Cameroon; b University of Maroua, National Advanced School of Engineering, Department of Agriculture, Livestock and By-Products, P. O. Box 45, Maroua, Cameroon; c University of Buea, Faculty of Science, Department of Biochemistry, P. O. Box 63, Buea, Cameroon; d University of Dschang, Faculty of Science, Department of Biochemistry, P. O. Box 67, Dschang, Cameroon

**Keywords:** *Polypterus bichir bichir*, Cooking treatment, Proximate composition, Lipid oxidation, Mineral content

## Abstract

The objective of this study was to evaluate the effect of different cooking and smoking treatments on proximate composition, lipid quality and mineral content of *Polypterus bichir bichir*, a fish consumed in Far-North Region of Cameroon. Results revealed that the proximate composition was significantly (P < 0.05) affected by treatments: except boiling, all the others treatment reduced significantly (P < 0.05) moisture content of fish while lipid and protein were significantly increased. After processing, the free fatty acids, peroxide and thiobarbituric acid reactive substances values **(**TBARS) were increased. Iodine value of all treated samples was significantly (P < 0.05) decreased. The combined treatments (frying + boiling and smoking + boiling) negatively affected the lipid quality of fish. Boiling caused significant losses in the mineral contents of fish while smoking treatment led to an important increase of its mineral contents. Steaming appeared to be the best processing method for cooking fish concerning the lipid stability.

## 1 Introduction

Hunger and malnutrition affect millions of people across the globe. In sub-Saharan Africa, about 210 million people lack adequate access to food (FAO, 2002). The growth of the population in this part of the globe puts more pressure on natural resources and more people will probably become food insecure, thereby lacking access to sufficient amounts of safe and nutritious food for normal growth, development, an active and healthy life (Pretty, 1999).

Fish represents an important food source for many food-insecure people. Regarding beneficial amounts of protein, lipids as well as essential micronutrients, fish and sea products have a high nutritional value. When compared to land living animals, fish are a rich source of protein and have a lower caloric density, a high content of omega-3 long chain polyunsaturated fatty acids (PUFAs) (Tocon and Metian, 2013). Consumption of fish and fish products has a positive health effect, especially with the decreased risk of coronary heart and cardiovascular diseases, decreased inflammatory disease and prevention of cancer (Calder, 2004; Lund, 2013). It has been established that docosahexaenoic acid (DHA) is very important for the maintenance of optimal pre and post-natal growth and development (Innis et al., 1999), so during pregnancy and neonatal period an optimal diet containing fish and sea food is very necessary for neural development of children. Eicosanoids are synthesized from n-3 PUFAs and have immunosuppressive properties (Calder, 2001). For many years, the positive effects of fish and seafood on human nutrition have been attributed to their high content of n-3 PUFAs. Today, many researchers proved that the main health-benefiting effects of fish and seafood consumption are also linked to other nutrients presents in these foods. Fish, fish products and seafood are excellent sources of high quality protein: bioavailability of protein from fish is approximately 5–15% higher than that from plant sources. The fish muscles contain myofibrillar proteins, sarcoplasmic proteins, connective tissue, stroma proteins, polypeptides, nucleotides and non-protein nitrogen compounds (Zmijewski et al., 2006). These foods have a well balanced amino acid composition. Taurine, another amino acid present in fish protein have similar anti-inflammatory effects as the long chain n-3 PUFAs. This molecule may also play an important role in health, for example, limiting the complication of type 2 diabetes and decreasing glucose, insulin and insulin resistance (Madani et al., 2012). Fish and fish products contain high content of vitamin D3 and B12, the major minerals present are calcium, phosphorus, iodine and selenium. They might provide significant amount of vitamin A, iron and zinc to consumers (Lund, 2013). Consumption of fish due to their high content of nutrients can help to reduce the risk of both malnutrition and non communicable diseases which may co-occur when high energy intake is combined with lack of balanced nutrition (Andew, 2001).

The nutritional values of fish are not constant. They are related to the life cycle of the fish, the temperature, salinity, and fatty acid composition of fish diet (Bandara et al., 2004). Danae et al. (2010) also reported that the types and levels of fatty acids founds in fish vary with species, age, size, reproduction stage, season, geographical location and diet.

In Cameroon and especially in the Far-North Region, many species of fish are present and can be used to fight against food insecurity and ameliorate nutritional status of population. Between these fish species, *Polypterus bichir bichir* is underutilized. This fish of the Polypteridae family is a common freshwater fish throughout the Nile and the White Nile in Sudan (Bailey, 1994) and live in various areas in Africa. *Polypterus bichir bichir* is a strictly carnivorous bottom-feeding fish and has a subcylindrical body. It has 63 to 70 scales in lateral line, 14 to 18 dorsal fin rays and 11 to 13 pelvic fin rays (Elagba, 2013). The nutritional value of these fish species is not well known.

The data of consumption of raw fish in Cameroon is meanwhile rare and information about nutritional content of raw fish may have limited value for a conclusion on their food quality (Al-Saghir et al., 2004). Boiling, grilling, frying, smoking and roasting are applied to food in different ways to improve its hygienic quality by the inactivation of pathogenic microorganisms, to enhance its flavour and taste, and increase shelf life (Porkorny, 1999). Nutritional evaluation of several species of marine and freshwater fishes has been reported (Weber et al., 2008; Tenyang et al., 2013, 2017; Akinwumi, 2014). It is known that during heat treatments, the physicochemical property of fish undergoes significant changes. The fat content of fish and meat especially, combined with a specific cooking methodology is among the factors that mostly affect the final quality of fish product (Serrano et al., 2007). Bognar (1998) reported that during cooking, digestibility is increased due to protein denaturation in food, but the content of thermolabile compounds, fat-soluble vitamins or polyunsaturated fatty acids is often reduced. The long chain PUFAs, such as eicosapentaenoic acid (EPA) and docosahexaenoic acid (DHA) are considered to be especially susceptible to oxidation during heat treatment and other culinary treatments (Candela et al., 1998; Tenyang et al., 2017). However, Candela et al. (1998) reported that in some fish species in certain types of cooking, it was found that the EPA and DHA contents remained unchanged. *Polypterus bichir bichir* which is present in the Far North of Cameroon are unknown by consumer and there is a general lack of information concerning their nutritional value. More information about lipid characteristics during treatment will be helpful to build accurate standards for fish lipids and create a more nutritional food through culinary processing. This study therefore aims to investigate the effect of boiling, frying and smoking, which are some common processing methods used in Cameroon on the proximate composition, lipid oxidation and mineral content of this freshwater fish consumed in the Far North Region of Cameroon.

## 2 Material and methods

### 2.1 Fish samples and pre-treatment

**Figure 1:**
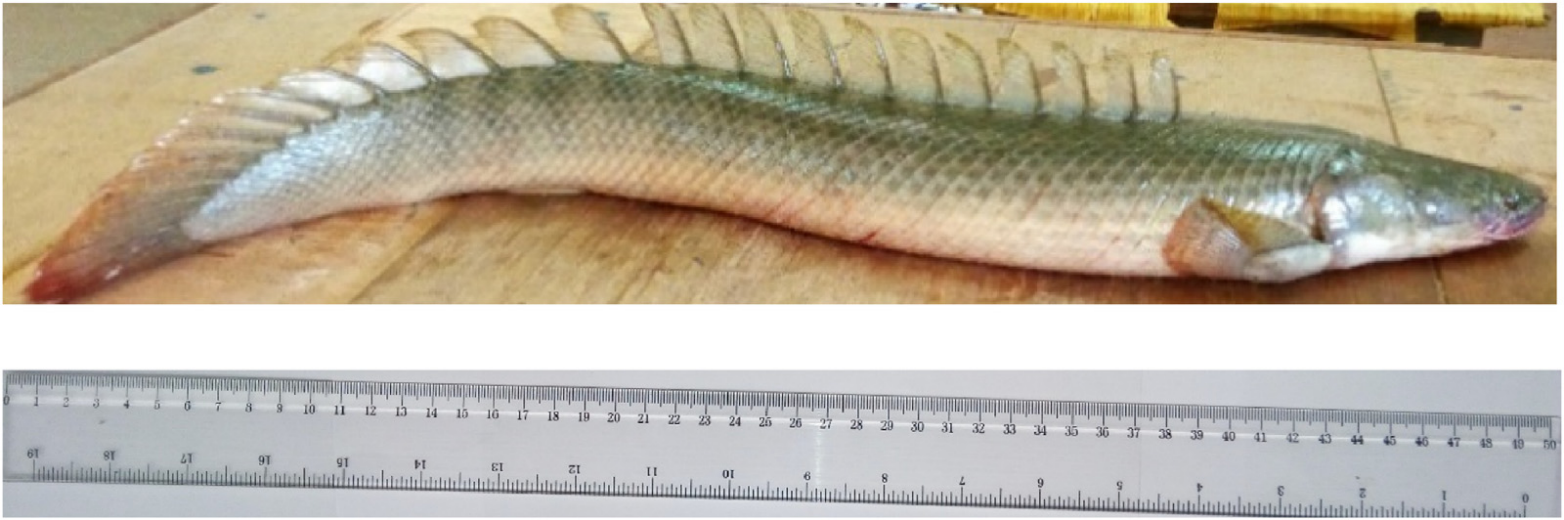
*Polypterus bichir bichir*.

Twenty one samples of *polypterus bichir bichir* (Figure 1), were collected from ponds in the locality of Maga (Division of Far-North Region of Cameroon) in July 2019. The mean weight and length of the fish were respectively 746 ± 11.3 g and 50.3 ± 6.30 cm. The collected fishes, were first transferred alive in boxes containing water to the Laboratory of Animal Biology for identification and later to the Food Biochemistry Laboratory of the University of Maroua. In the lab, they were killed by freezing at −20 °C for 24 h. Fish treatment and care was in accordance with the guidelines of the Cameroonian Bioethics Committee (Reg No FWA-IRB00001954) and following NIH-Care and Use of Laboratory Animals Manual (8th Edition). The protocol was approved by the Ethic Committee of Faculty of Sciences of the University of Maroua. After killing, they were washed with tap water to remove adhering mucus. Before treatment, the fishes were dipped into boiling water for 5 min to facilitate their scaling. The scaled fresh fish were prepared using a handling process, i.e. eviscerating, beheading and washing. The fish were subsequently randomly divided into 7 homogeneous groups of 3 fish each. One group was kept fresh-raw and used as control. The other 6 were subjected to cooking and smoking process.

### 2.2 Chemicals

Starch, potassium iodine, acetic acid, carbone tetrachloride, sodium thiosulfate, ethanol 95°, Wijs reactive, phenophtalein were procured from HiMedia Laboratories Pvt. Ltd (Mumbai, India). Hexane, chloroform, standard solutions for atomic absorption spectrometry (calcium, magnesium, sodium, potassium, iron, zinc, manganese, copper, phosphorus (purity 99.99%) were purchased from Courtage Analyses Services (Mont Saint-Aignan, France). All reagents used in the analysis were of laboratory grade.

### 2.3 Cooking and smoking treatments

#### 2.3.1 Boiling

Boiling treatment was performed at approximately 98 °C (water temperature) for 15 min. Whole fish samples (500 g) were boiled in 2 L of water using a domestic stainless-steel pot on a hot plate. immediately after boiling, the mean core temperature of *Polypterus bichir bichir* samples was 92 ± 3 °C.

#### 2.3.2 Frying and frying combined to ebullition

Whole samples of *Polypterus bichir bichir* were fried for 15 min in Palmor oil (palmitic acid: 36.69%, oleic acid: 47.3%, and linoleic acid: 9.25%) at an initial temperature of 150 °C in domestic pans of 3 L capacity. The oil was used once with the food/oil ratio being 250 g/L. After frying, the fishes were drained for about 4 min and then, one part of the fried fish was boiled in water for 10 min at 98 °C (water temperature).

#### 2.3.3 Steaming

Fish samples were placed in the stainless steel steamer above a stainless steel pot of boiling water (1 L) and cooked with the lid on for 15 min. The fish samples after cooking were placed on absorbent paper towels. The temperature of boiled water was 98 °C.

#### 2.3.4 Smoking and smoking combined to ebullition

The whole raw fish were spread out on smoking trays. The trays were then stacked on smoking oven fired with hard wood, at temperature greater than 70 °C. The smoking took 8 h to obtain a dry smoked product. Some smoked fish were then boiled in water for 10 min at 98 °C.

## 3 Analytical methods

The samples of raw, cooked and smoked *Polypterus bichir bichir* were immediately homogenised and used to determine proximate composition and mineral contents. The lipid oxidations were accessed using the free fatty acid, iodine, peroxide values and thiobarbituric acid reactive substances (TBARS).

### 3.1 Proximate analysis of fish

Proximate composition analyses of raw, cooked and smoked fish were done in triplicate for moisture, ash, lipid, and protein. Moisture content was determined by drying fish in oven at 103 °C until a constant weigh was achieved according to the AOAC procedures 925.40 (AOAC, 1990). Ash content was determined by incineration sesame fish at 550 °C according to the AOAC procedures 942.05 (AOAC, 1990). Total lipid was extracted from the muscle tissues using the Bligh and Blighet Dyer (1959) method. The lipid content was gravimetrically determined. Nitrogen (N) content was determined using micro-Kjeldahl method, according to AOAC procedures 984.13 (AOAC, 1990), the protein content was calculated as N x 6.25.

### 3.2 Chemical analyses of fish oil

#### 3.2.1 Measurement of free fatty acid

Free fatty acid (FFA) content was determined according to the method of AFNOR (1981). The fish oil sample (1 g) was dissolved in 100 ml of ethanol and some drop of phenolphthalein were added as an indicator and swirled vigorously. The mixture was then titrated with potassium hydroxide (0.1 M). The FFA was expressed as % oleic acid.

#### 3.2.2 Analysis of iodine value

The iodine value (IV) in the fish oil samples was determined using the Wijs method, as described by O’keefe and Pike (2010). The IV was expressed as grams of iodine absorbed per 100 g sample (g I_2_/100 g).

#### 3.2.3 Peroxide value

Peroxide value (PV) was determined according to the method described by AFNOR (1981). The fish oil sample (1 g) was treated with a 25 mL mixture of organic solvent (chloroform: acetic acid, 2:3). The mixture was then shaken vigorously, followed by the addition of 1 mL of saturated potassium iodide solution. The mixture was kept in the dark for 5 min. Distilled water (75 mL) was added to it and the mixture was shaken for 1 min. In the mixture, 0.5 mL of starch solution (1%, w/v) was added as an indicator. The peroxide value was determined by titrating the iodine liberated from potassium iodide with standardized 0.01 N sodium thiosulfate solution. The PV was expressed in milliequivalents of O_2_/kg of lipid. These determinations were performed in triplicate.

#### 3.2.4 Measurement of thiobarbituric acid reactive substance (TBARS)

The secondary oxidation products were evaluated using the thiobarbituric acid reactive substance method as described by Drapper and Hadley (1990). The results were expressed as mg of malondialdehyde (MDA) per kg of sample.

### 3.3 Mineral determination of defatted fish flour

For the determination of minerals, defatted fish flour was ashed at 550 °C and the ash boiled with 10 ml of 20% HCl in a beaker and then filtered into a 100 ml standard flask to determine the mineral content. Calcium (Ca), magnesium (Mg), sodium (Na), potassium (K), iron (Fe), zinc (Zn), and copper (Cu) were determined by atomic absorption spectrometer (Varian 220FS Spectr AA, Les Ulis, France). Phosphorus (P) was determined colorimetrically using the vanado molybdate, according to AOAC procedure 965.17 (AOAC 1999). Reference sample from parts of the daily routine in the laboratory was used for quality control. Certified reference material 1570a was purchased from the National Institute of Standard and Technology (Gaithersburg, USA). After initial standardisation of techniques during a pilot study, the samples were treated identically. Mineral contents of the samples were determined from calibration curves of standards minerals. All samples were analysed in triplicate.

## 4 Statistical analysis

Data were analysed by a one-way analysis of variance (ANOVA) and comparison was made between species and treatments. The statistical treatment was performed at a 5% significance level using the Statistical Package of Social Science (SPSS 16.0 version).

## 5 Results and discussion

### 5.1 Effects of treatments on proximate composition of *Polypterus bichir bichir*

The proximate composition of raw, cooked and smoked *Polypterus bichir bichir* is presented in Table 1. Moisture content varied from 44.5 to 73.3%. The Lowest moisture content was characteristic of fried samples (44.5%) and maximum values were found for raw and boiled samples (∼73.3%). Except in boiled sample, an explicit tendency to decrease the moisture content in all samples due to heat treatment occurred. Moisture content of raw *P.b. bichir* obtained in this study was lower than 79%, value noted by Weber et al. (2008) in raw catfish fillets. Steamed samples compared to boiled ones presented lower moisture content. The same trend was noted between fried and smoked samples. Fried and smoked samples when submitted to boiling, their moisture content significantly increased (P < 0.05). Decrease in moisture content during cooking were also noted by others researchers (Abroumand, 2014; Benguendouz, 2018).

**Table 1:** Effects of cooking and smoking on proximate composition of *Polypterus bichir bichir*.

	Moisture (%)	Ash (%)	Lipid (%)	Protein (%)	Energy value (Kcal/100 g)
Raw	73.3 ± 0.17^a^	2.19 ± 0.08^e^	5.74 ± 0.59^d^	16.4 ± 0.06^e^	117 ± 7.17^f^
Boiled	72.8 ± 1.50^a^	2.13 ± 0.10^e^	6.27 ± 0.17^d^	17.2 ± 0.43^e^	125 ± 0.27^f^
Fried	44.5 ± 1.01^e^	5.25 ± 0.22^a^	15.3 ± 0.42^a^	36.0 ± 0.71^a^	281 ± 1.33^a^
Fried + Boiled	59.5 ± 0.59^c^	3.75 ± 0.10^c^	9.86 ± 0.89^c^	25.4 ± 1.28^c^	190 ± 4.09^c^
Smoked	51.2 ± 0.08^d^	4.66 ± 0.02^b^	12.4 ± 0.11^b^	31.2 ± 0.64^b^	236 ± 2.22^b^
Smoked + Boiled	61.1 ± 0.69^c^	3.55 ± 0.05^c^	8.44 ± 0.45^c^	25.2 ± 0.99^c^	177 ± 0.15^d^
Steamed	67.7 ± 0.45^b^	2.69 ± 0.04^d^	8.32 ± 1.00^c^	21.0 ± 0.25^d^	159 ± 11.3^e^

Values are presented as means ± standard deviation (n = 3). Mean values in the same column with different superscript letters are significantly different (P < 0.05).

Except for boiled sample, the ash content of others cooked samples increased significantly (p < 0.05) and fried samples were found to exhibit a higher ash content (5.25%), followed by smoked samples (4.66%). The boiling of fried and smoked fish decreased significantly (p < 0.05) their ash content. Similar trends were observed by Tiwo et al. (2016) when evaluating the effect of cooking methods on proximate composition of four freshwater fish species. Increase in ash content during processing could be due to the water dehydration as explained by Salan et al. (2006). The high ash content observed in this study in cooked samples may also be due to inclusion of bones as edible parts in fish samples, which would lead to high ash value. Boiling of fried and smoked fish samples decreased significantly their ash content. The total lipid and proteins of raw *Polypterus bichir bichir* were respectively 5.74% and 16.4%. The protein content (16.4%) of raw sample can be assumed to be of high dietary quality, being an animal source protein (WHO, 2007). The crude lipid of fish is constituted principally of unsaturated fatty acids, which have important physiological functions according to the previous studies (Calder, 2004; Lund, 2013). Except in the boiled sample, the lipid and protein contents were significantly (P < 0.05) affected by all cooking methods. These nutrients increased significantly (P < 0.05) after processing. Benguendouz (2018) have mentioned the same trend when studying the effect of cooking on proximate composition of *Sardina pilchardus*. This can be explained by the reduction in moisture content which occurs during certain processing. Arias et al. (2003) explained that the decrease in moisture content in cooked fish has been described as the change that makes the lipid and protein increased.

Compared to others sample, the fried fish was found to have higher lipid content. This may be correlated to the oil absorption during the frying process. Tenyang et al. (2013) reported that increase of lipid content in food during frying can be explained by the oil penetration into the food after partial water loss by evaporation. These results are in agreement with those of Golpolipour et al. (2019) who demonstrated that the amount of carp lipid and proteins significantly (P < 0.05) increases with frying. Boiling treatment affected negatively the lipid content of fried and smoked *Polypterus bichir bichir.* Steamed *Polypterus bichir bichir* compared to boiled one had high lipid and protein contents. Decrease in these nutrients during boiling treatment may be linked to their losses in cooking water. These results are similar to most results of lipids changes in fish during different cooking methods, such as frying, boiling and steaming (Nieva-Echevarria et al., 2017; Golpolipour et al., 2019). The energy value of raw and cooked fish is presented in Table 1. Since lipid content is the most important component from the energy content point of view, the increase in the lipid content of products caused an increase in the energy content of foods. It could be noticed that energy value of fish sample increased significantly during processing except boiling treatment. Boiling has no effect on energy value of *Polypterus bichir bichir*. Nevertheless, all the others treatments increased significantly the energy value of fish. The higher energy value (281.7 kcal/100 g) was found to fried sample, following by smoked sample, fried + boiled sample, smoked + boiled sample and the lower energy value was found to steamed sample. Steamed fish compared to boiled fish had significantly (P ˃ 0.05) higher energy value, which may be due to the less lipid contained in boiled sample. A significant higher energy value found in fried fish compared to others treated samples is a consequence of higher content of protein and lipid. These results are in agreement with those of Seena et al. (2006) who noted that the calorie content increased significantly during roasting of seeds of mangrove legume.

### 5.2 Effect of cooking and smoking on lipid quality of *Polypterus bichir bichir*

#### 5.2.1 Acid value of oils extracted from processed fish

**Figure 2:**
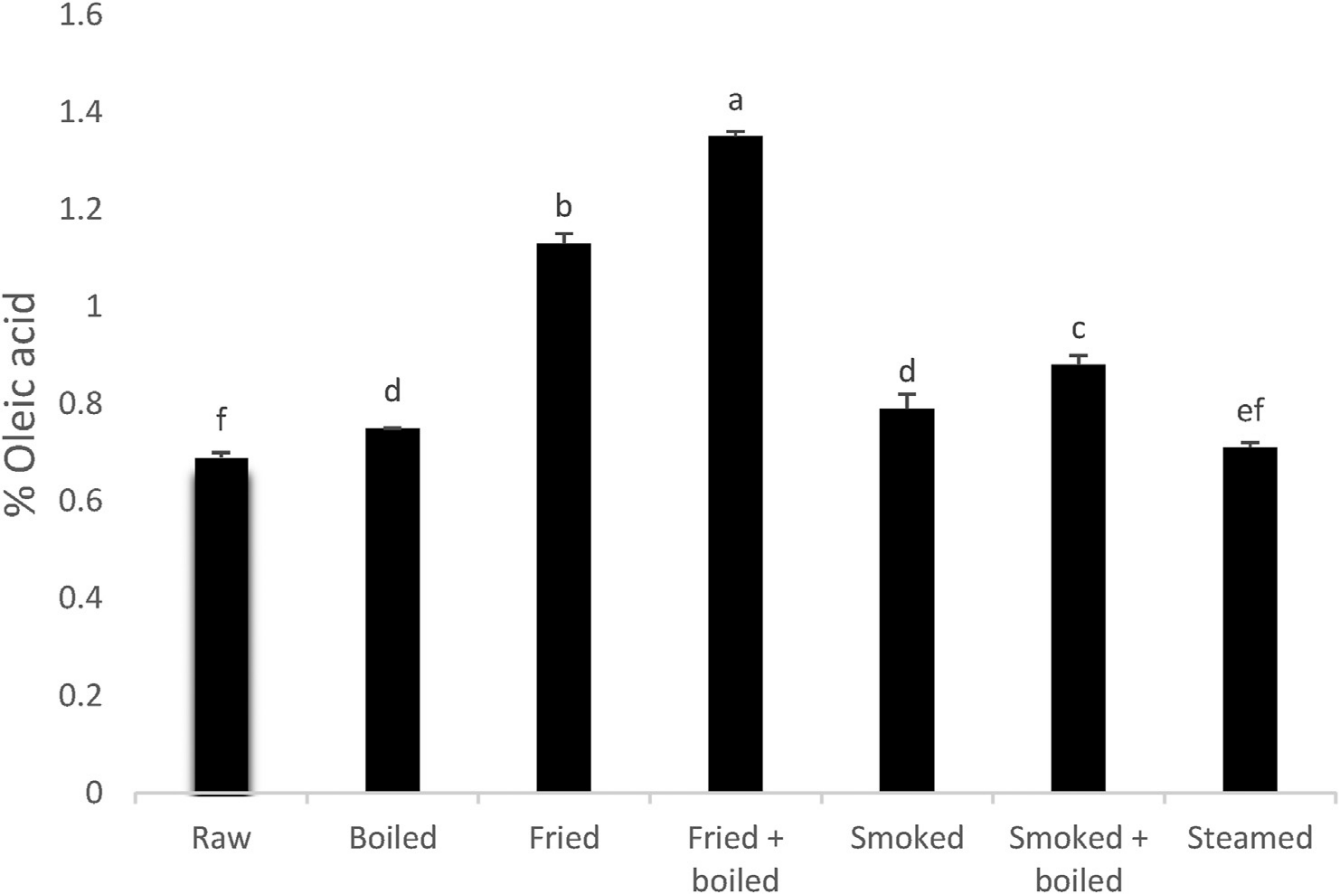
Changes in acid value of *Polypterus bichir bichir* during processing. Results are means ± standard error (n = 3). Bars that have no common letters are significantly different (p < 0.05).

Expressed as a percentage of oleic acid in oil, acid value allows the determination of free fatty acids (FFAs). The FFAs are produced due to the hydrolysis of triglycerides and phospholipids in the oil. The hydrolysis process can be promoted moisture content. The better information on that acidity of glycerides should be obtained from acid value, which takes into account the contribution of all the constituent fatty acids in the oil and fat, and that this parameter is the preferred quality control parameter used by paint manufacturers to monitor the concentrations of acids in resins (O’keefe and Pike, 2010). As presented in Figure 2, the FFAs content of raw sample was 0.69% oleic acid. This value is lower than 2.86% and 3.73% oleic acids recorded by Weber et al. (2008) and Tenyang et al. (2018) in raw *Rhamdia quelen* and *Clupea harengus* respectively. The initial FFAs in raw fish oil is due to the action of endogenous enzymes present in the viscera prior to acidification. Except steamed samples, FFAs content of *Polypterus bichir bichir* increased significantly (p < 0.05) with the cooking methods and fried + boiled fish was found to present the highest value (1.35% oleic acid). Boiled and smoked samples presented no significant (P > 0.05) difference in FFAs content. The fried compared to smoked samples had higher FFAs content. Boiling of fried and smoked samples increased significantly (p < 0.05) their acid value. Increased in FFAs during boiling may be linked to heating water that catalyses the hydrolysis of ester bonds of triglycerides and phospholipids and releases FFAs in oil. According to the study of Hernandez et al. (1999), the heat treatment can increase the FFAs of oils. During frying, oils develop acidity due not only to oxidation and decomposition of intermediate oxidation products, which is highly relevant, but also due to hydrolysis. These observations are in disagreement with those of Weber et al. (2008) who observed a decrease of FFAs in boiled catfish oil. A very high temperature applied during frying is of responsible to high breakdown of ester bonds of triglycerides present in fish oil by thermolysis. Increase in FFAs during frying is also in discordance with the results mentioned by Al-Shagir et al. (2004) who noted the decreased of FFAs in pan fried salmon fillets. The FFAs contents obtained in this work are within the range specified for edible oils which is 2.89% oleic acid, as given by FAO/WHO (2009). Consequently, all these cooked samples were in good quality.

#### 5.2.2 Iodine value of oils extracted from processed fish

**Figure 3:**
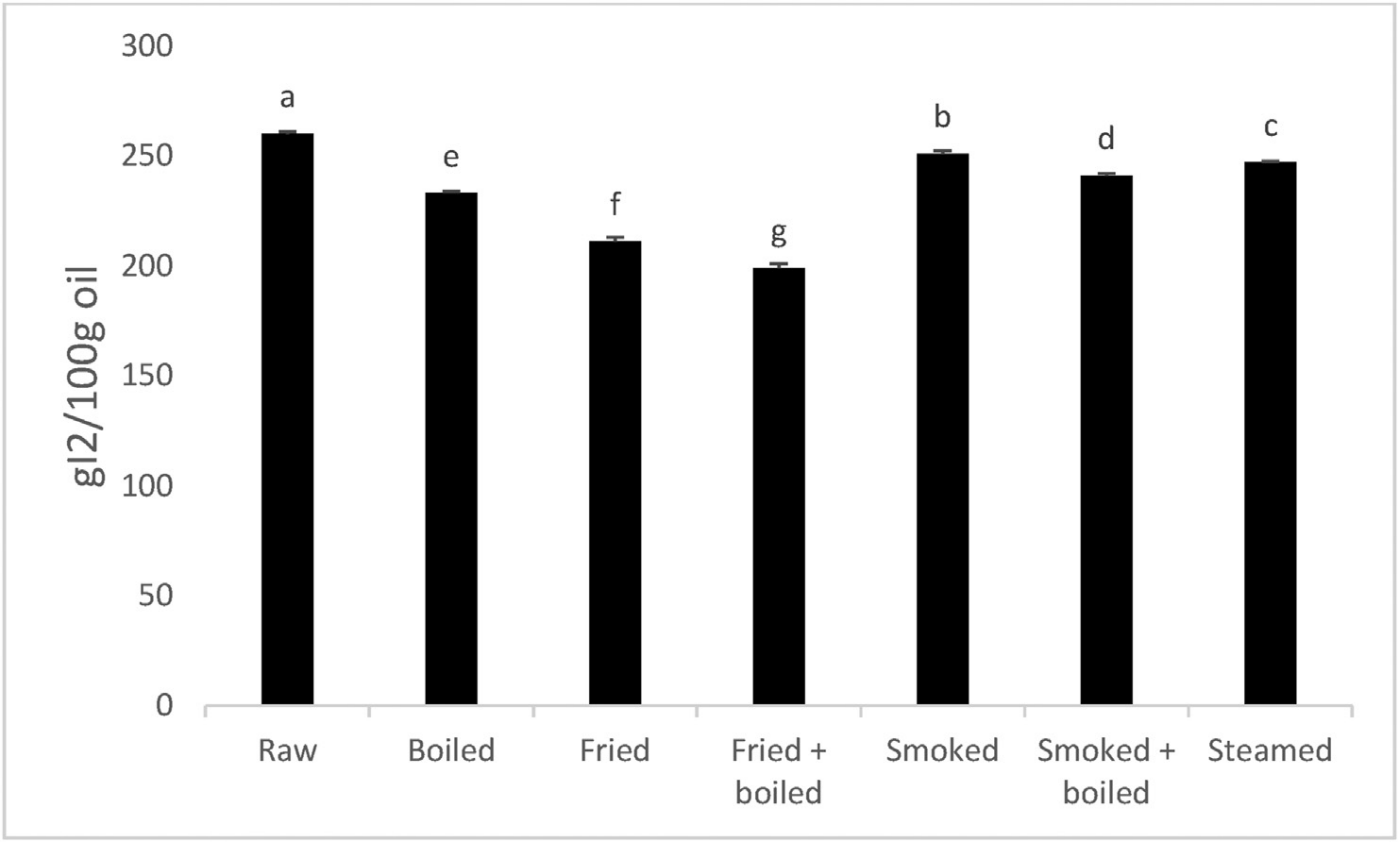
Changes in iodine value of *Polypterus bichir bichir* during processing. Results are means ± standard error (n = 3). Bars that have no common letters are significantly different (p < 0.05).

Fatty fish contains more than 60% unsaturated fatty acids (UFAs). These fatty acids are susceptible to lipid oxidation. During processing, UFAs are attacked by free radicals, which results in the decrease of double bonds (Nyam et al., 2003). To measure the degree of unsaturation of oil and fat, iodine value (IV) is used. The IV is defined as the number of grams of iodine that will react with the double bonds in 100 g of fat. A high IV indicates that the sample has greater number of double bonds (Baiao and Lara, 2005). Changes in IV of *Polypterus bichir bichir* during culinary processing are presented in Figure 3. The IV of *Polypterus bichir bichir* oil was in the range 193–260 gI_2_/100 g oil. Raw samples were found to have a higher IV (260 gI_2_/100 g oil). This obtained value is higher than those noted by Sharina and Jumat (2006) in Malaysia for raw Menhaden and Aji-Aji fish oils (188 and 110 gI_2_/100g oil, respectively). Yasushi et al. (2005) when analysing the IV of fish oil obtained 166 and 155 gI_2_/100 g oil in raw salmon and sardine oils. These values are also lower as those obtained in this study. The noted variation of IV may be due to differences in specie, growing environment and fish diet (Danae et al., 2010). The higher IV of raw *Polypterus bichir bichir* oil could be explained by their high level of highly UFAs such as eicosapentaenoic, docosahexaenoic and arachidonic acids. During processing, a progressive decrease in IV was observed and fried + boiled treatment showed the lowest IV (199 gI_2_/100 g oil). Steamed fish compared to boiled samples has a higher IV. Combination of two cooking methods decreased significantly (p < 0.05) the IV of fish oil samples. These observations are in agreement with the study of Tenyang et al. (2017) who noted a significant decrease of IV in smoked *Clupea harengus*. The decrease in IV noted during processing may be due to the changes in fatty acid taking place with heat. The high temperature can be the cause of lipid oxidation of oils (Orthoefer et al., 1996; Tenyang et al., 2017). These explain the highest decrease in IV in fried samples. The protective role of smoke produce during smoking process resulted in a small decrease in IV in smoked fish samples.

#### 5.2.3 Peroxide value of oils extracted from processed fish

**Figure 4:**
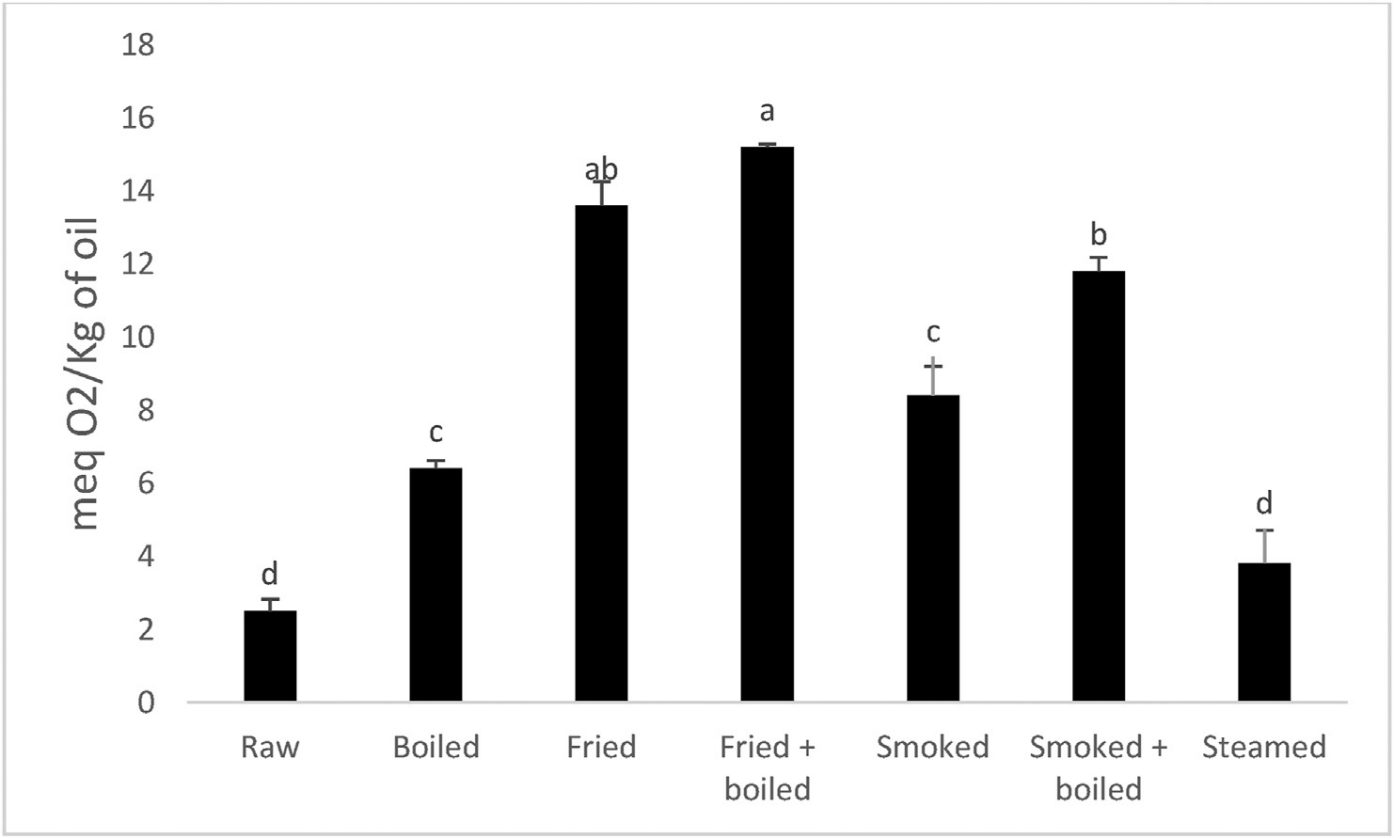
Changes in peroxide value of *Polypterus bichir bichir* during processing. Results are means ± standard error (n = 3). Bars that have no common letters are significantly different (p < 0.05).

The first stage of the oxidation process is characterised by the production of hydroperoxides, a primary oxidation product, which is usually measured as the peroxide value (Juntachote et al., 2007). Oil and fat are oxidized during processing via autoxidation in which triplet oxygen and singlet oxygen react with free fatty acids (FFAs). High PV in oils makes them more susceptible to rancidity and having a shorter life. Figure 4 shows the results of the effects of cooking and smoking treatments on PV of *Polypterus bichir bichir* oils. There was a significant (P < 0.05) difference in PV of all cooked and smoked samples. Peroxide value of samples ranged between 2.50 and 15.22 meqO_2_/kg of oil. The lowest PV was obtained in raw and steamed oil samples, while the highest was obtained in fried + boiled *Polypterus bichir bichir* oil. There was no significant (p > 0.05) difference in PV between raw and steamed sample oils. The same conclusion is found between boiled and smoked sample oils. Fried and fried + boiled sample oils showed no significant (p > 0.05) difference in PV. Our results of fried samples are in accordance with those reported by Tenyang et al. (2013) who observed an increase in the PV of fried catfish. The increase of PV noted during processing is due to the accumulation of hydroperoxides as a result of free radical attacking the unsaturated fatty acids (Nyam et al., 2013; Tenyang et al., 2017). Deep frying also promoted high lipid oxidation differences between fresh and fried samples, and may be explained by actual autoxidation, which is the main mechanism of oxidation of oil (Nyam et al., 2013). Therefore, immersing fish samples in oil likely increases lipid levels in their composition, causing greater lipid oxidation in the samples subjected to deep frying process. Variation in PV for all treatments may be due to processing applied and heat temperature. The peroxide values of all the processed samples were within the maximum acceptable value of 15 meq O_2_/Kg of oil as recommended by Codex Alimentarius commission (Codex Alimentarius commission, 1999). Due to their low PV (3.81 meq/kg of oil), steamed fish oil provides a best index of oxidative quality.

#### 5.2.4 Thiobarbituric acid reactive substances value (TBARS) of oils extracted from processed fish

**Figure 5:**
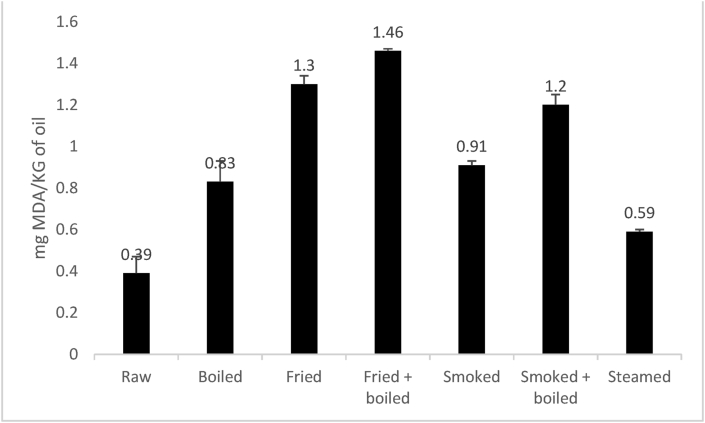
Effect of cooking and smoking methods on TBARS value changes of *Polypterus bichir bichir* during processing.

During processing, at high temperature, the initial hydroperoxides formed exist only transiently and are rapidly decomposed into various volatile and non-volatile products (Saguy and Dana, 2003). To evaluate the secondary oxidation state of oils and fats, generally, TBARS value is used. Malonaldehyde (MDA) which is a secondary lipid oxidation product obtained during processing contributes to the off-flavour of the oil. Measure of MDA concentration can serve as oxidative damage index. The production of secondary lipid oxidation products in food contributes to the deterioration of their nutritive value. The TBARS values of oil extracted on cooked and smoked *Polypterus bichir bichir* are presented in Figure 5. A significant increase in the TBARS value was observed in all samples. The lower TBARS value (0.39 mg/kg) was recorded in raw samples, while the highest TBARS values were found in fried and fried + boiled samples (1.46 mg/kg). No significant (p > 0.05) difference was observed in the TBARS value between boiled and smoked samples. Steamed fish compared to boiled one had the lower TBARS value. The increase in the TBARS values during processing is probably due to the fact that heating could develop oxidative processes and increase MDA levels in fish (Al-Saghir et al., 2004). The high temperature used may be acting as a good catalyst of the formation of hyddroperoxides and to their decomposition into secondary oxidation products among such as aldehydes, ketones and alcohols (Ceriane and Meirelless, 2007). The results of boiled samples are in agreement with those of Weber et al. (2008) who observed an increased in TBARS value in boiled salmon fish. However, for fried samples, the TBARS values in the current study are in discordance with the observation of Weber et al. (2008) who rather noted a decrease in TBARS level in fried salmon fish. The most significant (p < 0.05) increase in TBARS value in fried samples could be explained by the migration of lipid oxidation products from the frying oil into the fish. Notably, the fry oil used in this study contains UFAs, and Ansorena et al. (2010) have well established that susceptibility to lipid oxidation enhances with the increased unsaturation of the molecule. Al-Kahtani et al. (1996) reported that meat products that have less than 3 mg MDA/kg is considered as good for human consumption. Concerning the samples evaluated in this study, they were suitable for consumption and steamed fish was found to present the good oxidative quality due to their low TBARS value.

### 5.3 Effect of cooking and smoking on mineral contents of *Polypterus bichir bichir*

The change in mineral contents of raw, cooked and smoked *Polypterus bichir bichir* are presented in Table 2. For raw sample, calcium (Ca) was quantitatively the most important macroelement, followed by phosphorous (P) and potassium (K). magnesium was the less important macrroelemnt. Iron in this study was found to be quantitatively the most abundant microelement followed zinc (Zn) and copper (Cu). The present results of major elements (Ca, P, K) concentration in raw *Polypterus bichir bichir* were similar to those reported by Tenyang et al. (2020) from the tissue of raw *Chrysichthys nigrodigitatus* from Maga lake in Cameroon, but different from those obtained by Gokoglu et al. (2004) in rainbow trout (*Oncorhynchus mykiss*). The Ca content of raw *Polypterus bichir bichir* was found to be 1064 mg/100 g. This value is lower than 5171 mg/100g, value obtained by Tenyang et al. (2020) in raw *C. nigrodigitatus*.

**Table 2:** Mineral composition of raw, cooked and smoked *Polypterus bichir bichir* (mg/100 g).

	Ca	Mg	P	Na	K	Cu	Zn	Fe
Raw	1064 ± 12^a^	52.3 ± 0.28^bc^	867 ± 5.7^b^	69.1 ± 0.03^b^	809 ± 9.90^c^	5.52 ± 0.01^b^	5.65 ± 0.01^c^	15.1 ± 0.15^a^
Boiled	952 ± 10.5^b^	44.9 ± 1.27^e^	738 ± 4.24^e^	53.2 ± 0.10^e^	497 ± 4.24^e^	3.90 ± 0.06^f^	4.30 ± 0.01^d^	12.9 ± 0.10^b^
Fried	696 ± 14.1^d^	50.5 ± 0.03^cd^	681 ± 3.49^f^	77.1 ± 0.07^a^	829 ± 7.07^c^	5.98 ± 0.02^a^	5.40 ± 0.00^c^	12.1 ± 0.04^bc^
Fried + Boiled	616 ± 8.48^e^	48.4 ± 0.05^d^	661 ± 8.49^f^	61.1 ± 0.14^d^	517 ± 15.6^e^	4.86 ± 0.00^d^	3.91 ± 0.07^e^	10.4 ± 0.07^c^
Smoked	976 ± 15.6^b^	55.7 ± 0.03^a^	928 ± 7.78^a^	69.5 ± 0.41^b^	924 ± 2.83^a^	4.37 ± 0.01^e^	6.84 ± 0.05^a^	15.2 ± 0.25^a^
Smoked + Boiled	744 ± 7.07^c^	53.3 ± 0.14^ab^	834 ± 2.83^c^	67.1 ± 0.00^c^	880 ± 4.95^b^	3.89 ± 0.04^f^	5.41 ± 0.10^c^	13.2 ± 0.28^b^
Steamed	1004 ± 15.0^ab^	52.3 ± 1.48^bc^	805 ± 11.3^d^	61.1 ± 0.08^d^	602 ± 14.2^d^	5.19 ± 0.01^c^	6.42 ± 0.21^b^	16.7 ± 1.40^a^

Values are presented as means ± standard deviation (n = 3). Mean values in the same column with different superscript letters are significantly different (P < 0.05).

In this study, processing had a significant influence on minerals content of *Polypterus bichir bichir*. Except steamed samples, the Ca content of all processed samples in this study significantly (P < 0.05) decreased and fried + boiled samples presented the lowest Ca content (616 mg/100 g). No significant (p > 0.05) difference in Ca content was found between raw and steamed *Polypterus bichir bichir*. The results obtained in this work in smoked samples are in disagreement with the report of Tenyang et al. (2020) who noted an increase in Ca content in smoked *C. nigrodigitatus*. However, for fried and boiled samples, Gokoglu et al. (2004) in cooked rainbow trout noted similar trends. Similarly, Tiwo et al. (2016) when analysing the Ca content of boiled *Clarias Gariepinus* and *Cyprinus carpio* observed the decrease of Ca content.

The content of Mg which is a component of bone in raw *Polypterus bichir bichir* was 52.3 mg/100 g. This result is within the FAO range of 4.5–452 mg/100 g. However, the value is higher than 17 mg/100 g, value reported by Wheaton and Lawson (1985). Smoking significantly (P < 0.05) increased the Mg content of *Polypterus bichir bichir* compared to other treatments. No significant (P > 0.05) difference was found between the Mg contents of raw, fried, smoked + boiled and steamed samples. Boiling process decreased significantly (P < 0.05) the content of Mg of these fish samples and their values were still very low (44.9 mg/100 g).

The phosphorus content of raw *Polypterus bichir bichir* was found to be 867 mg/100 g. This value is lower than 1375 mg/100 g, reported by Mogabe et al. (2015) in some small fish consumed in Botswana. Smoking treatment increased significantly (P < 0.05) the P content of *Polypterus bichir bichir* while all others treatments decreased it significantly (P < 0.05). The lower value of 661 mg/100 g was found in fried + boiled samples. Steamed compared to boiled samples had the higher P content. Increase in P content during smoking process, is in disagreement with the report of Kiczorowska et al. (2019) who noted a decrease in the amount of P in smoked fish. In agreement with the results of this study, Ersoy and Ozeren (2009) found lower P content in fried and boiled fish fillets as compared to fresh one.

The levels of sodium (Na) are presented in Table 2. This macroelement is very important for muscle functioning. The data showed that their content in raw fish was 69.1 mg/100 g. During processing, except smoking and frying treatments, all other treatments decreased significantly (P < 0.05) the Na content of *Polypterus bichir bichir* and the boiled samples were found to have the lowest Na content (53.2 mg/100 g). Decrease in Na content observed in boiled samples is in line with results mentioned by Gokoglu et al. (2004) in boiled rainbow trout.

Raw *Polypterus bichir bichir* contained 809 mg/100 g of potassium (K). This value is higher than 319–429 mg/100 reported by Jim et al. (2017) in Zimbabwe in fishes from three ecosystems. The increase in K content was significant (P < 0.05) for fried, smoked, smoked + boiled samples. Smoked samples compared to other treatments showed the highest content of K (924 mg/100 g). Boiled fish compared to steamed fish samples exhibited lower amount of K.

Copper (Cu) content of raw *Polypterus bichir bichir* was 5.52 mg/100 g (Table 2). This content was affected differently by the cooking methods applied. Frying treatment increased significantly (P < 0.05) the Cu content while other treatments decreased significantly (P < 0.05) this value. The lower Cu contents were found in boiled and smoked + boiled samples (3.89 mg/100 g). Decrease in Cu content during frying is in line with the reports of Gokoglu et al. (2004) in fried rainbow trout. However, Joyce et al. (2016) observed an increase of Cu content in fried fish.

The levels of zinc (Zn) of *Polypterus bichir bichir* are presented in Table 2. Smoking and steaming methods increased significantly (p < 0.05) the amount of Zn in *Polypterus bichir bichir* and the highest value was obtained in smoked fish (6.84 mg/100 g). Raw, Fried and smoked + boiled samples showed the same Zn content. The lowest Zn content (3.91 mg/100 g) was obtained in fried + boiled *Polypterus bichir bichir*.

In the body, iron (Fe) is an important microelement for a number of physiological functions, but most important for transporting oxygen around body. Its deficiency causes anaemia which is a public health problem in the world, especially in low income countries (Galani et al., 2020). The content of Fe in raw *Polypterus bichir bichir* was 15.3 mg/100 g. This value is lower than 21 mg/100 g, value reported by Gokoglu et al. (2004) in raw rainbow trout. Smoking and steaming methods have no significant (P > 0.05) effect on Fe content of *Polypterus bichir bichir*. Boiling, frying, frying + boiling, and smoking + boiling methods decreased significantly (P < 0.05) the Fe content of *Polypterus bichir bichir* and the lowest amount was found in the fried + boiled samples (10.42 mg/100 g). These results obtained in this study are in discordance with those of and Tenyang et al. (2020) who noted an increase of Fe content in some smoked fish.

Variation in mineral contents observed in the present work may be due to changes induced by culinary treatments. Ackurt (1991) found that the processing and cooking methods had little or no effect on the mineral contents, but Djopnang et al. (2018) reported that the amount of mineral in some fish samples was affected by cooking methods.

## 6 Conclusion

*Polypterus bichir bichir*, a fish commonly present in Far North Region of Cameroon was a good nutritional source especially for ash, lipid and proteins. All the treatments applied, except boiling treatment, enhanced the ash, lipid and protein contents of *Polypterus bichir bichir*. Frying in palmor at 150 °C for 15 min was the best cooking method in terms of increase in nutritive and energy value of *Polypterus bichir bichir*. Acid, peroxide and TBARS values of *P.b. bichir* after processing increased significantly while iodine value decreased. The combined treatments (frying + boiling and smoking + boiling) negatively affected the lipid quality of fish. Steaming for 15 min at 98 °C appeared to be the best processing method for cooking these fish concerning the lipid stability. Boiling caused significant losses in mineral contents of fish while smoking resulted in important increases in their mineral contents, in particular Ca, Mg, P, K, and Zn. The results from this work are very useful for the cooking of *Polypterus bichir bichir* and can enhanced its utilization.

## Declarations

### Author contribution statement

Noel Tenyang: Conceive and designed the experiment; Performed the experiment; Analyzed and interpreted the data; Contributed reagents, materials, analysis data; Wrote the paper.

LUDOVINE ATEUFACK MAWAMBA; Hilaire Macaire Womeni: Performed the experiment; Analyzed and interpreted the data; Wrote the paper.

ROGER PONKA; BERNARD TIENCHEU: Contributed reagents, materials, analysis of data.

ABAZIDI MAMAT: Performed the experiments.

### Funding statement

This research did not receive any specific grant from funding agencies in the public, commercial, or not-for-profit sectors.

### Data availability statement

Data included in article/supp. material/referenced in article.

### Declaration of interest’s statement

The authors declare no conflict of interest.

### Additional information

No additional information is available for this paper.
